# Influence of Lifestyle Factors on Mammographic Density in Postmenopausal Women

**DOI:** 10.1371/journal.pone.0081876

**Published:** 2013-12-09

**Authors:** Judith S. Brand, Kamila Czene, Louise Eriksson, Thang Trinh, Nirmala Bhoo-Pathy, Per Hall, Fuat Celebioglu

**Affiliations:** 1 Institution of Medical Epidemiology and Biostatistics, Karolinska Institutet, Nobels Väg 12A, Stockholm, Sweden; 2 National Clinical Research Centre, Level 3, Dermatology Block, Hospital Kuala Lumpur, Jalan Pahang, Kuala Lumpur, Malaysia; 3 Julius Centre University of Malaya, Faculty of Medicine, University of Malaya, Lembah Pantai, Kuala Lumpur, Malaysia; 4 Department of Clinical Science and Education, Södersjukhuset (KI SÖS), S1. Sjukhusbacken 10, Stockholm, Sweden; National University of Ireland Galway, Ireland

## Abstract

**Background:**

Mammographic density is a strong risk factor for breast cancer. Apart from hormone replacement therapy (HRT), little is known about lifestyle factors that influence breast density.

**Methods:**

We examined the effect of smoking, alcohol and physical activity on mammographic density in a population-based sample of postmenopausal women without breast cancer. Lifestyle factors were assessed by a questionnaire and percentage and area measures of mammographic density were measured using computer-assisted software. General linear models were used to assess the association between lifestyle factors and mammographic density and effect modification by body mass index (BMI) and HRT was studied.

**Results:**

Overall, alcohol intake was positively associated with percent mammographic density (*P* trend  = 0.07). This association was modified by HRT use (*P* interaction  = 0.06): increasing alcohol intake was associated with increasing percent density in current HRT users (*P* trend  = 0.01) but not in non-current users (*P* trend  = 0.82). A similar interaction between alcohol and HRT was found for the absolute dense area, with a positive association being present in current HRT users only (*P* interaction  = 0.04). No differences in mammographic density were observed across categories of smoking and physical activity, neither overall nor in stratified analyses by BMI and HRT use.

**Conclusions:**

Increasing alcohol intake is associated with an increase in mammography density, whereas smoking and physical activity do not seem to influence density. The observed interaction between alcohol and HRT may pose an opportunity for HRT users to lower their mammographic density and breast cancer risk.

## Introduction

Percent mammographic density refers to the relative amount of fibroglandular to fat tissue in the breast and is a major risk factor for breast cancer with risk being 4–6 fold higher in women with extremely dense breasts (>75%) compared to women having fatty or non-dense breasts (<5%) [1]. Because of its strong association with breast cancer, it is plausible that factors influencing mammographic density may also contribute to breast cancer risk. Hence, identification of such factors will not only improve our understanding of breast cancer etiology, but may also provide an avenue for cancer prevention in the instances when the risk factors are modifiable.

A large number of studies have examined the effect of lifestyle factors on mammographic density in postmenopausal women, but except for hormone replacement therapy (HRT) results have not been entirely consistent. For instance, while most studies have reported a positive association between alcohol intake and mammographic density [2]–[6], the association was not always significant [5], [6] and in some studies no effect of alcohol on density was found [7]–[9]. Data on the effect of smoking are limited and even more conflicting with some studies reporting an inverse relation [2], [10], [11] and others showing a null association [8], [12]. Evidence regarding the impact of physical activity is also inconsistent. An inverse association has been reported [13]–[15], but in the majority of studies no difference in mammographic density with physical activity levels was found [16].

Most studies thus far have focused on percent density as a measure of mammographic density, but the effect of lifestyle factors may be better understood by examining its individual components: the absolute dense and non-dense area. Furthermore, there is some evidence that lifestyle factors may influence breast cancer risk differently according to body size [17], [18] and HRT use [18]–[20], but only few studies to date have considered these factors as possible effect modifiers when studying breast density [3], [14], [15].

To clarify the effect of lifestyle factors on mammographic density we conducted a cross-sectional study in postmenopausal women. In this study, we examined associations between alcohol, smoking and physical activity with different mammographic measures (percent density, absolute dense and non-dense area) while taking potential interactions with body mass index (BMI) and HRT use into account.

## Methods

### Study population

The current analysis was based on postmenopausal control subjects within the CAHRES study, a nationwide breast cancer case-control study among women born in Sweden aged 50 to 74 years at time of enrolment (1 October 1993–31 March 1995). Details on the study design and data collection have been described previously [21]. A total of 4118 control subjects were randomly selected from the general population according to the expected age distribution of breast cancer cases (in 5-year intervals) using the National Population Register which holds data on national registration number, name, address and place of birth of all Swedish residents. Among the invited women, 3454 agreed to participate (response rate: 82%). All participants provided written informed consent and the study was approved by the ethical review board at Karolinska Institutet and the regional ethical review boards in Stockholm, Gothenburg, Umeå, Linköping, Malmö-Lund and Uppsala.

For the present study, we excluded women with a previous cancer (other than nonmelanoma skin cancer and cervix cancer in situ (N = 94)), premenopausal women (N = 151) and women with unknown menopausal status (N = 73), leaving 3136 eligible control subjects. Menopause was defined as the age at the last menstrual period or the age at bilateral oophorectomy if at least 1 year prior to study entrance. Details on mammography retrieval have been described elsewhere [22]. Briefly, we sought to retrieve mammograms from all CAHRES participants using the Swedish national registration numbers. During 2006 and 2007, all mammography screening units and radiology departments conducting screening mammography throughout Sweden were visited. Mammograms were successfully collected for 1702 control subjects (54.3%). Women with mammography data were no different from those without in terms of age, BMI, age at menarche, age at menopause and parity. However, women with mammograms were slightly older at first birth (24.8 years vs. 24.3 years), were more likely to have breastfed (82.6% vs. 78.7%) and tended to use HRT more often (42.0% vs. 38.7%).

Since mammographic density changes substantially around menopause [23], we excluded women with premenopausal mammograms (N = 18). We further excluded women with incomplete data on lifestyle factors (N = 537), leaving 1147 postmenopausal women for the analysis. The median interval between the date of mammography and study entrance was 61 days.

### Mammographic density

The medio-lateral oblique (MLO) view, which is the routine view for breast cancer screening in Sweden, was used to assess mammographic density. Film mammograms were digitized using an Array 2905 HD Laser Film Digitizer (Array Corporation, Tokyo, Japan), which covers a range of 0–4.7 optical density. Mammographic density was quantified using the computer-assisted software Cumulus, developed at the University of Toronto, Canada [24]. For each image, a trained observer (LE) set the appropriate gray-scale threshold levels defining the edge of the breast and distinguishing dense from non-dense tissue. The software calculates the total number of pixels within the entire breast area and the region identified as dense. The non-dense area was obtained by subtracting the dense area from the total breast area. The size of the dense and the non-dense areas in cm^2^ was determined by multiplying the number of pixels in the respective areas by the size of one pixel. Percent density was calculated by dividing the dense area by the total breast area and multiplying by 100. A 10% random sample was read twice by the same observer to assess the intra-observer reliability which was high (R^2^ = 0.92).

### Lifestyle factors

Participants were asked in detail about their life-course smoking history. Smoking status (current, former, never) was defined based on responses to the questions: ‘Have you ever been smoking regularly for more than 1 year or have you ever smoked more than 100 cigarettes? ‘and ‘Were you smoking one year ago?’. In addition, ever smokers were asked to recall the number of cigarettes they smoked per day during each decade of life and at the time of study entry. Based on this information, smoking dose, average smoking duration and cumulative smoking exposure in pack-years were estimated. One pack-year was defined as smoking 20 cigarettes/day for 1 year. All smoking measures were modeled as categorical variables: smoking dose (1–5, 6–10 and >10 cigarettes/day), smoking duration (1–10 years, 11–30 years, >30 years) and cumulative smoking exposure (1–10, 11–20, 21–30, >30 pack-years).

Current alcohol intake was assessed by asking about the average monthly consumption of beer, wine and hard liquor over the preceding year. Total alcohol intake in grams per month was calculated by summing the alcohol content from each alcoholic beverage. In the algorithm we used the following beverage-specific glass volumes and alcohol contents: regular beer 200 ml–5.6 g, strong beer 200 ml–9.0 g, wine 100 ml–8.9 g, strong wine 40 ml–6.4 g and liquor 40 ml–12.8 g. The total monthly consumption in grams was then divided by 30 to obtain the alcohol intake in g/day. Non-drinkers were defined as women who reported no consumption of any type of alcoholic drinks and drinkers were categorized according to their daily alcohol consumption (0.1–4.9 g/day, 5.0–9.9 g/day and ≥10 g/day).

Physical activity was assessed based on the amount of leisure time spent each week on physical exercise/sports during three time periods (childhood, from ages 18–30 and during the year preceding study enrolment) using predefined categories (never, <1 hour/week, 1–2 hours/week, >2 hours/week).

### Covariates

The questionnaire also included information on reproductive factors, exogenous hormone use and anthropometry. Participants were asked to report their age at first menstruation and use of oral contraceptives and HRT. Age at menopause was defined as either age at last menstruation or age at bilateral oophorectomy. Participants were also asked about their age at first birth and whether they had ever breastfed. Body mass index was calculated based on the self-reported height (cm) and weight (kg).

### Statistical analyses

First, women were categorized according to quartiles of percent density and differences in participant characteristics were tested using linear trend tests for continuous variables and Chi-square linear trend tests for categorical variables. We then used general linear models to examine the associations between lifestyle factors and the different mammographic measures (percent density, dense and non-dense area). We fitted two models to adjust the analyses for potential confounders. The first model was adjusted for age at mammography (years) only. In the multivariable model we further adjusted the analyses for BMI (kg/m^2^), age at menarche (years), age at menopause (years), OC use (ever, never), HRT use (current, former, never) and a combined variable for parity and age at first birth (nulliparous, 1 child age at first birth <25 years, 1 child age at first birth ≥25 years, 2 children age at first birth <25 years, 2 children age at first birth ≥25 years, ≥3 children age at first birth <25 years, ≥3 children age at first birth ≥25 years). In the multivariable model, we also mutually adjusted the analyses for other lifestyle factors: smoking (current, former, never), current alcohol intake (non-drinker, 0.1–4.9 g/day, 5.0–9.9 g/day and ≥10 g/day) and physical activity in recent years (never, <1 hour/week, 1–2 hours/week, >2 hours/week). Eighteen percent of the women had missing values on one or more covariates. Because missing values were likely to be missing at random, and to avoid loss in efficiency, missing values were imputed by means of multiple imputation [25] using the ICE package in STATA [26]. All variables of the multivariable adjusted model were included in the imputation model and 10 imputation sets were created.

To examine potential effect modification by BMI and HRT, we added multiplicative interaction terms to the models (i.e. the product of alcohol (in categories) and potential effect modifiers (BMI continuous and HRT in categories). Since the effect of HRT on mammographic density is acute and diminishes within a few weeks after cessation [27], we distinguished between current and ever use when modeling HRT as an effect modifier.

Two density measures (percent density and dense area) were square root transformed prior to analyses to approximate the normal distribution. For ease of interpretation, the estimated means and confidence intervals were back-transformed to the original scale. Data were analyzed using STATA version 12.0 (Stata Corp., College Station, TX, USA).

## Results

Characteristics of the study population are summarized in **Table 1**. The mean age at mammography was 63.7 (standard deviation 6.1) years and approximately 75% of the women had a percent density of less than 20%. Almost one-third of the women were current smokers and 16% were not physically active in recent years. More than half of the population reported drinking alcohol, but only a small proportion drank ≥10 g/day. As expected, age and BMI were strongly inversely associated with percent density. Nulliparous women had a higher percent density, and so did women who ever used oral contraceptives and women using hormone replacement therapy. Older ages at menarche and first birth were also positively associated with percent density, whereas density was lower among women who ever breastfed. Percent density did not differ according to smoking status, but women with dense breasts were more likely to drink alcohol and to be physically active in recent years.

**Table 1:** Characteristics of the study population, overall and by quartiles of percent density.

	Total (N = 1147)	Quartiles of percent density (%)				
		Q1 (<4.08) (N = 287)	Q2 (4.08–9.99) (N = 287)	Q3 (10.00–20.20) (N = 287)	Q4 (>20.2%) (N = 286)	*P* trend
Age at mammography (years), mean (SD)	63.7 (6.1)	64.6 (5.7)	63.6 (6.1)	63.5 (6.3)	62.6 (6.2)	0.001
BMI (kg/m^2^), mean (SD)	25.6 (4.0)	27.4 (4.3)	26.2 (3.9)	25.1 (3.5)	23.8 (3.1)	<0.001
Age at menarche (years), mean (SD) †	13.5 (1.4)	13.4 (1.3)	13.4 (1.5)	13.5 (1.3)	13.9 (1.5)	0.001
OC use (ever),% (N) †	35.0 (400)	30.3 (87)	34.0 (97)	36.7 (105)	38.8 (111)	0.03
Age at menopause (years), mean (SD)	50.1 (4.1)	50.1 (4.1)	49.8 (4.3)	50.1 (4.0)	50.4 (3.8)	0.23
Parity,% (N)						<0.001
0	10.5 (120)	6.6 (19)	9.8 (28)	10.1 (29)	15.4 (44)	
1	18.7 (215)	18.5 (53)	18.5 (53)	19.2 (55)	18.9 (54)	
2	35.8 (411)	31.0 (89)	41.5 (119)	36.2 (104)	34.6 (99)	
≥3	35.0 (401)	43.9 (126)	30.3 (87)	34.5 (99)	31.1 (89)	
Age at first birth (years), mean (SD) * †	24.9 (4.8)	24.2 (4.8)	24.4 (4,5)	24.8 (4.8)	26.2 (4.9)	<0.001
Breastfeeding (ever),% (N) * †	83.7 (848)	86.5 (211)	85.1 (217)	84.0 (215)	79.5 (205)	0.03
HRT use,% (N)						<0.001
Never	54.6 (626)	62.0 (178)	57.5 (165)	54.0 (155)	44.8 (128)	
Former	14.8 (170)	15.0 (43)	15.7 (45)	16.4 (47)	12.2 (35)	
Current	30.6 (351)	23.0 (66)	26.8 (77)	29.6 (85)	43.0 (123)	
Smoking status,% (N)						0.10
Never	59.6 (684)	61.7 (177)	60.6 (174)	60.3 (173)	55.9 (160)	
Former	18.7 (214)	19.5 (56)	18.5 (53)	17.8 (51)	18.9 (54)	
Current	21.7 (249)	18.8 (54)	20.9 (60)	22.0 (63)	25.2 (72)	
Physical activity recent years,% (N)						0.02
Never	16.3 (187)	17.4 (50)	18.5 (53)	17.1 (49)	12.2 (35)	
Less than 1 hr per week	16.0 (183)	18.5 (53)	16.7 (48)	13.2 (38)	15.4 (44)	
1–2 hrs per week	31.8 (365)	30.0 (86)	32.4 (93)	32.1 (92)	32.9 (94)	
More than 2 hrs per week	35.9 (412)	34.2 (98)	32.4 (93)	37.6 (108)	39.5 (113)	
Alcohol intake,% (N)						<0.001
Non-drinker	48.2 (553)	55.8 (160)	54.0 (155)	41.1 (118)	42.0 (120)	
0.1–4.9 g/day	38.5 (442)	34.8 (100)	35.5 (102)	41.5 (119)	42.3 (121)	
5.0–9.9 g/day	8.6 (99)	5.9 (17)	6.3 (18)	13.2 (38)	9.1 (26)	
≥10 g/day	4.6 (53)	3.5 (10)	4.2 (12)	4.2 (12)	6.6 (19)	

Abbreviations: BMI  =  body mass index; OC  =  oral contraceptive; HRT  =  hormone replacement therapy. * In parous women only. † Percentage of women with missing values on age at menarche (9.2%), OC use (0.3%), age at first birth (10.5%) and breastfeeding (11.7%).

**Table 2** shows the adjusted means of the different mammographic measures by alcohol intake and smoking habits. Estimates obtained after multiple imputation were similar to those in complete cases. After multivariable adjustment, a weak positive association between alcohol intake and percent density was found (*P* trend  = 0.07). The adjusted mean percent density in heavy drinkers (>10 g/day) was 13.0% (95% CI 10.1–16.2%) compared to 10.7% (95% CI 9.9–11.6%) in non-drinkers. Analyses of individual components showed that the positive trend between alcohol and percent density was mainly driven by a smaller non-dense area in alcohol drinkers (*P* trend  = 0.02).

**Table 2:** Age and multivariable adjusted means of mammographic density measures by alcohol intake and smoking habits.

		Mammographic measures, mean (95% CI)					
	N	Percent density (%) *		Dense area (cm^2^) *		Nondense area (cm^2^)	
		Model 1	Model 2	Model 1	Model 2	Model 1	Model 2
Alcohol intake							
Non-drinker	553	10.12 (9.25–11.02)	10.70 (9.86–11.59)	17.00 (15.58–18.48)	17.56 (16.12–19.07)	159.6 (154.9–164.3)	154.7 (150.9–158.4)
0.1–4.9 g/day	442	12.02 (10.97–13.13)	11.71 (10.74–12.72)	18.88 (17.21–20.62)	18.59 (16.97–20.29)	146.3 (141.0–151.5)	148.8 (144.8–152.9)
5.0–9.9 g/day	99	12.94 (10.69–15.41)	11.96 (9.94–14.18)	20.57 (16.99–24.48)	19.52 (16.10–23.26)	141.4 (130.3–152.5)	147.9 (139.3–156.5)
≥10 g/day	53	15.28 (11.97–18.99)	12.96 (10.10–16.18)	21.70 (16.75–27.29)	19.75 (15.08–25.05)	126.5 (111.3–141.7)	144.1 (132.3–156.0)
*P* trend		<0.001	0.07	0.01	0.21	<0.001	0.02
							
Smoking status							
Never	684	11.08 (10.25–11.94)	11.51 (10.72–12.33)	18.12 (16.79–19.50)	18.50 (17.18–19.88)	154.1 (149.8–158.4)	150.6 (147.3–153.9)
Former	214	10.92 (9.48–12.45)	10.36 (9.05–11.75)	17.57 (15.29–20.02)	16.61 (14.42–18.96)	150.8 (143.2–158.5)	152.0 (146.2–157.9)
Current	249	12.25 (10.81–13.77)	11.52 (10.22–12.90)	19.08 (16.84–21.47)	18.87 (16.67–21.21)	144.2 (137.1–151.4)	152.9 (147.3–158.4)
*P* trend		0.23	0.79	0.57	0.98	0.02	0.48
Smoking duration †							
1–10 years	91	11.45 (9.19–13.96)	12.16 (9.95–14.61)	19.05 (15.38–23.12)	19.32 (15.62–23.41)	155.3 (143.9–166.7)	147.7 (138.9–156.5)
11–30 years	200	10.27 (9.73–12.92)	10.91 (9.48–12.44)	17.82 (14.78–20.43)	17.35 (14.97–19.91)	146.5 (138.8–154.2)	147.8 (141.9–153.7)
>30 years	171	12.91 (11.13–14.82)	12.96 (11.27–14.78)	19.34 (16.60–22.29)	19.77 (17.00–22.75)	139.6 (131.3–147.9)	142.1 (135.7–148.5)
*P* trend		0.26	0.40	0.78	0.67	0.03	0.25
Smoking dose at entry							
1–5 cigarettes/day	47	13.63 (10.29–17.44)	13.01 (9.78–16.70)	20.73 (15.63–26.54)	18.87 (14.54–25.55)	144.6 (137.8–152.0)	143.1 (129.8–156.4)
6–10 cigarettes/day	71	11.48 (8.97–22.86)	11.52 (9.04–14.31)	18.04 (14.15–22.39)	18.51 (14.44–23.08)	150.1 (141.8–158.5)	146.5 (135.7–152.0)
>10 cigarettes/day	98	11.77 (9.58–14.18)	12.02 (9.84–14.41)	18.43 (15.04–22.15)	18.57 (15.08–22.43)	137.8 (124.6–151.0)	146.4 (137.3–155.6)
*P* trend		0.46	0.72	0.55	0.78	0.71	0.72
Packyears of smoking †							
1–10	208	11.32 (9.81–12.94)	11.21 (9.78–12.73)	17.79 (15.40–20.35)	17.56 (15.20–20.09)	147.1 (139.5–154.7)	147.4 (142.1–152.6)
11–20	133	12.07 (10.13–14.17)	11.96 (10.14–13.93)	19.78 (16.66–23.16)	19.65 (16.56–23.02)	148.4 (138.9–157.8)	147.9 (140.1–155.8)
21–30	86	11.27 (8.95–13.86)	11.63 (9.40–14.11)	17.36 (13.73–21.41)	17.83 (14.17–21.92)	144.1 (132.2–156.0)	132.1 (119.6–144.6)
>30	35	16.73 (12.42–21.69)	16.94 (12.77–21.70)	22.61 (16.33–29.90)	23.31 (16.88–30.79)	130.7 (112.3–149.2)	134.2 (118.6–149.9)
*P* trend		0.12	0.06	0.38	0.23	0.20	0.10

There was no significant difference in mammographic density between smokers and non-smokers. Neither current nor past smokers had altered density measures (**Table 2**). Although current smokers and long-term smokers had a smaller non-dense area, these associations became non-significant after adjustment for potential confounders (*P* trend  = 0.48 and *P* trend  = 0.25 respectively). Smoking dose was also not associated with any of the density measures. However, a positive trend of percent density with increasing pack-years was seen after multivariable adjustment (*P* trend  = 0.06), though inspection of individual components did not reveal a significant association.

Overall, no clear association between physical activity and mammographic density was found **(** **Table 3** **)**. Increasing levels of recent activity were associated with a higher percent density and smaller non-dense area in age-adjusted models, but these associations were no longer significant after multivariable adjustment (*P* trend  = 0.66 and 0.40 for percent density and non-dense area respectively). Physical activity levels during childhood and from age 18 to 30 years were also not related to any of the mammographic measures **(** **Table 3** **)**.

**Table 3:** Age and multivariable adjusted means of mammographic density measures by physical activity.

		Mammographic measures, mean (95% CI)					
	N	Percent density (%) *		Dense area (cm^2^) *		Nondense area (cm^2^)	
		Model 1	Model 2	Model 1	Model 2	Model 1	Model 2
Physical activity recent years							
Never	187	10.14 (8.67–11.73)	11.25 (9.81–12.80)	17.27 (14.85–19.86)	18.30 (15.86–20.92)	160.5 (152.4–168.7)	151.2 (144.9–157.4)
Less than 1 hr per week	183	10.20 (8.71–11.82)	10.82 (9.39–12.35)	17.23 (14.79–19.87)	17.71 (15.29–20.31)	159.9 (151.7–168.1)	154.3 (148.0–160.6)
1–2 hrs per week	365	11.61 (10.47–12.81)	11.39 (10.35–12.48)	18.37 (16.57–20.26)	18.15 (16.41–19.98)	150.3 (144.5–156.1)	152.2 (147.8–156.6)
>2 hrs per week	412	12.06 (10.97–13.21)	11.45 (10.46–12.47)	18.98 (17.26–20.79)	18.48 (16.82–20.22)	144.3 (138.8–149.7)	149.3 (145.2–153.5)
*P* trend		0.02	0.66	0.19	0.79	<0.001	0.40
Physical activity childhood							
Never	192	10.07 (8.62–11.64)	10.86 (9.46–12.36)	16.51 (14.17–19.02)	17.25 (14.92–19.76)	156.0 (147.9–164.1)	149.5 (143.3–155.7)
Less than 1 hr per week	139	10.65 (8.91–12.55)	10.44 (8.85–12.16)	16.73 (13.99–19.71)	16.62 (13.96–19.51)	149.8 (140.3–159.3)	152.4 (145.2–159.6)
1–2 hrs per week	319	12.08 (10.84–13.39)	11.76 (10.63–12.94)	19.25 (17.28–21.32)	18.90 (17.00–20.89)	149.0 (142.7–155.2)	150.9 (146.1–155.6)
>2 hrs per week	493	11.49 (10.51–12.51)	11.44 (10.54–12.37)	18.72 (17.15–20.35)	18.67 (17.15–20.25)	151.5 (146.5–156.5)	152.1 (148.2–155.9)
*P* trend		0.11	0.35	0.09	0.21	0.49	0.58
Physical activity 18–30 years							
Never	197	9.67 (8.26–11.18)	10.60 (9.23–12.07)	15.96 (13.69–18.40)	17.01 (14.71–19.47)	158.1 (150.2–166.2)	151.8 (146.7–157.9)
Less than 1 hr per week	175	11.20 (9.60–12.93)	11.31 (9.82–12.90)	17.60 (15.08–20.32)	17.77 (15.30–20.43)	149.3 (140.8–157.7)	149.4 (143.0–155.8)
1–2 hrs per week	382	12.15 (11.00–13.35)	11.44 (10.41–12.51)	19.28 (17.47–21.17)	18.30 (16.57–20.11)	148.0 (142.3–153.7)	151.0 (146.6–155.3)
>2 hrs per week	392	11.38 (10.29–12.52)	11.51 (10.50–12.57)	18.68 (16.93–20.52)	18.99 (17.27–20.79)	152.1 (146.5–157.7)	152.4 (148.1–156.7)
*P* trend		0.08	0.35	0.06	0.17	0.33	0.71

* Percent density and dense area were square-root transformed for analysis and the values shown are on a back-transformed scale. † In ever smokers only.

Model 1: adjusted for age at mammography

Model 2: adjusted for age at mammography, BMI, age at menarche, parity and age at first birth (nulliparous, 1 child age at first birth <25 years, 1 child age at first birth ≥25 years, 2 children age at first birth <25 years, 2 children age at first birth ≥25 years, ≥3 children age at first birth <25 years, ≥3 children age at first birth ≥25 years), age at menopause, OC use (never, ever), HRT use (never, former, current), smoking (never, former, current), alcohol intake (non-drinker, 0,1–4,9 g/day, 5.0–9.9 g/day, ≥10 g/day), and physical activity recent years (never, less than 1 hr per week, 1–2 hrs per week, >2 hrs per week).

Interaction analyses showed that the effect of alcohol on percent density was modified by HRT (*P* interaction  = 0.06) (**Table 4**). In current HRT users, a significant positive association between alcohol intake and percent density was found (*P* = 0.01) with non-drinkers and heavy drinkers having an adjusted percent density of 12.4% (95% CI 10.3–14.6%) and 20.7% (95% CI 14.4–28.2%) respectively. By contrast, no association between alcohol and percent density was observed in non-current users (*P* trend  = 0.82). Since 1 standard drink equals 10 grams of alcohol, these results indicate that women using HRT and drinking more than 1 glass of alcohol per day have an 8% higher percent density on average than HRT users who are alcohol abstainers. The effect of alcohol on absolute dense tissue was also modified by HRT (*P* interaction  = 0.04). Again, the absolute dense area increased with increasing alcohol intake in current HRT users only (*P* trend  = 0.01). Notably, interactions with HRT were not significant when modeling ever instead of current use (**Table 4**). Associations with alcohol were not modified by BMI and for both smoking and physical activity no evidence of effect modification by BMI or HRT was found (data not shown).

**Table 4:** Multivariable adjusted means of mammographic density measures by alcohol intake, stratified by HRT use.

		Mammographic measures, mean (95% CI)			
		N	Percent density (%) *	Dense area (cm^2^) *	Nondense area (cm^2^)
Current HRT use	*Yes*				
	Non-drinker	138	12.36 (10.34–14.56)	19.53 (16.14–23.25)	146.1 (138.6–153.7)
	0,1–4,9 g/day	155	15.15 (13.10–17.34)	24.21 (20.75–27.94)	139.5 (132.6–146.4)
	5,0–9,9 g/day	36	16.95 (12.56–22.00)	26.47 (19.15–34.97)	128.2 (113.8–142.7)
	≥10 g/day	22	20.68 (14.36–28.17)	33.56 (22.81–46.39)	124.2 (105.1–143.4)
	*P* trend		0.01	0.01	0.01
	*No*				
	Non-drinker	415	9.90 (9.02–10.83)	16.43 (14.93–18.00)	158.4 (154.1–162.6)
	0,1–4,9 g/day	287	10.22 (9.16–11.34)	16.21 (14.45–18.08)	153.5 (148.4–158.5)
	5,0–9,9 g/day	63	9.61 (7.48–12.01)	16.10 (12.47–20.21)	157.3 (146.4–168.1)
	≥10 g/day	31	10.26 (7.20–13.86)	15.09 (10.23–20.90)	154.3 (138.8–169.7)
	*P* trend		0.82	0.74	0.35
					
	*P* interaction		0.06	0.04	0.11
Ever HRT use	*Yes*				
	Non-drinker	211	12.18 (10.60–13.88)	19.47 (16.80–22.33)	148.7 (142.7–154.7)
	0,1–4,9 g/day	227	13.86 (12.27–15.54)	22.03 (19.37–24.86)	141.3 (135.7–146.9)
	5,0–9,9 g/day	51	13.93 (10.69–17.61)	22.80 (17.28–29.07)	140.4 (128.6–152.3)
	≥10 g/day	32	15.32 (11.01–20.34)	24.23 (17.05–32.67)	134.8 (119.6–150.0)
	*P* trend		0.15	0.17	0.05
	*No*				
	Non-drinker	342	9.46 (8.54–10.43)	15.79 (14.22–17.44)	159.9 (155.1–164.6)
	0,1–4,9 g/day	215	10.02 (8.84–11.27)	15.88 (13.93–17.97)	155.2 (149.3–161.2)
	5,0–9,9 g/day	48	10.38 (7.91–13.19)	17.02 (12.84–21.79)	154.0 (141.4–166.6)
	≥10 g/day	21	11.21 (7.46–15.72)	16.38 (10.44–23.64)	154.0 (134.9–173.0)
	*P* trend		0.28	0.70	0.23
	*P* interaction		0.94	0.70	0.58

* Percent density and dense area were square-root transformed for analysis and the values shown are on a back-transformed scale.

Model 1: adjusted for age at mammography.

Model 2: adjusted for age at mammography, BMI, age at menarche, parity and age at first birth (nulliparous, 1 child age at first birth <25 years, 1 child age at first birth ≥25 years, 2 children age at first birth <25 years , 2 children age at first birth ≥25 years, ≥3 children age at first birth <25 years, ≥3 children age at first birth ≥25 years), age at menopause, OC use (never, ever), HRT use (never, former, current), smoking (never, former, current), alcohol intake (nondrinker, 0,1–4,9 g/day, 5.0–9.9 g/day, ≥10 g/day), and physical activity recent years (never, less than 1 hr per week, 1–2 hrs per week, >2 hrs per week).

## Discussion

To our knowledge this is the first population-based study examining the effect of multiple lifestyle factors on different mammographic measures in postmenopausal women. Our data suggest that mammographic density increases with increasing alcohol intake, whereas smoking and physical activity do not seem to influence density. Although the overall association between alcohol intake and percent density did not reach statistical significance, an interaction between alcohol and HRT was evident. In current HRT users, increasing alcohol intake was associated with a higher percent density, whereas no such association was found in non-current users. Further examination of the individual components revealed that increasing levels of alcohol intake are associated with both a larger dense and smaller non-dense area in current HRT users.

The effect of alcohol on breast cancer risk is well-established as previous studies have consistently demonstrated a linear dose-response relation between alcohol intake and breast cancer risk [28]. Also biologically, there are several plausible mechanisms through which alcohol could increase breast cancer risk including direct DNA damage, enhanced mammary gland susceptibility and elevated sex hormone levels [29]. Nevertheless, the available data on alcohol and mammographic density are not entirely consistent. Our finding of a positive trend of increasing mammographic density with increasing alcohol intake is consistent with most [2]–[6], but not all studies [7]–[9]. The null findings in some studies can in part be explained by small sample sizes [8], [9] and low average alcohol intake [7], [9], with the latter resulting in a smaller exposure range and lower power to detect an association. In addition, interactions with other lifestyle habits may account for some of inconsistent results. Indeed, we observed that the effect of alcohol was modified by HRT, with a positive association being significant in current HRT users only. An interaction with HRT has been reported previously by Yaghiyan et al. [16] who found that the association with alcohol was stronger in ever HRT users. However, in that study no distinction was made between current and past users. Interestingly, we found that the interaction did only hold for current use. This finding is not unexpected given that mammographic density is most strongly influenced by current HRT use [27] and the effect may thus attenuate when current and past users are combined. The observed interaction with current use is also in line with previous observations for breast cancer. Several studies have shown that the association between alcohol intake and breast cancer risk is particularly strong in current HRT users [19], [20], [30]. The exact mechanism underlying the synergistic effect of alcohol and HRT is not completely understood, but may reflect their shared influence on sex hormones. Alcohol and HRT are most strongly associated with hormone sensitive breast cancers [31], [32] and both have an impact on circulating estrogens levels [33], [34]. In the absence of HRT, alcohol causes a modest increase in endogenous estrogens [33], most likely through a stimulatory effect on the adrenal glands [35]. The effect of alcohol appears to be more pronounced in the presence of HRT, as a rapid three-fold increase in estrogen levels has been reported upon alcohol intake in HRT users [36]. The acuteness of this effect is indicative of an altered estrogen clearance rate in alcohol drinkers. Another interesting observation is that increasing levels of alcohol intake were not only associated with a greater dense area, but also a smaller non-dense area, suggesting a fat-reducing effect in peripheral tissues. In women, alcohol is primarily degraded through the microsomal ethanol oxidizing system which is a high energy demanding process [37]. Alcohol may also reduce body fatness by increasing the level of diet-associated thermogenesis [38]. Since we collected no information on different adipose measures it is unclear whether the observed decrease in non-dense area is due to a smaller amount of central body fat or reflects a dual effect of alcohol on breast density. Because the non-dense area has also been implicated in breast cancer risk [39], future studies are needed to disentangle the net effect of alcohol on breast fat.

Evidence on the effect of smoking on mammographic density is conflicting. While most studies in pre- and perimenopausal women have found a lower mammographic density in smokers [12], [40], results are inconsistent in postmenopausal women with either an inverse association [2], [10], [11] or no association [8], [12]. The reported inverse associations, however, need to be interpreted with caution, as effect estimates were modest and may result from residual confounding by body size given the strong association between BMI and smoking [40]. Overall, we could not identify a clear association between smoking status and mammographic density, although a positive trend of percent density with increasing pack-years was seen. This finding seems to be in line with recent data showing an increased breast cancer risk with cumulative smoking exposure [41], but given the lack of association with individual density components, further research is needed to determine the effect of long-term heavy smoking on mammographic density.

Our results for physical activity are in agreement with a recent systematic review [16], showing no effect of physical activity on mammographic density. Nonetheless, there is evidence for a role of physical activity in breast cancer risk. In a recent meta-analysis of prospective studies a linear relationship between physical activity levels and breast cancer risk was found, with risk being highest in vigorously active women [42]. Also, several biological mechanisms have been proposed including changes in body size, sex hormone levels and inflammatory markers. Our null findings may thus argue against mammographic density being a mediator in the physical activity-breast cancer relation, but we cannot exclude the possibility of subgroup effects. There are some indications that changes in mammographic density may only be detectable in conditions of vigorous activity and in women with a high BMI [16]. We and others [43] could not identify an interaction with BMI, but a number of studies have reported an inverse association between physical activity and mammographic density in overweight and obese women [13]–[15]. A potential modifying effect of BMI is also supported by physical activity trials showing a reduction in sex hormones levels in overweight postmenopausal women [44]. Furthermore, the apparent positive association between recent activity and breast density prior to BMI adjustment [43], [45], [46] underscores the strong negative confounding effect of BMI, which may have hampered the detection of an association. Therefore, further large-scale studies with subgroup analyses are needed to determine the effect of physical activity on mammographic density.

Strengths of our study are the population-based design, detailed information on potential confounders and measurement of mammographic density using a quantitative semi-automated method. As different mammographic measures were assessed, we were able to examine differential effects on relative and absolute density measures. Nevertheless, our study also had several limitations. While we had detailed information on smoking history, we could only examine rather crude measures for alcohol intake and physical activity. For this reason, we were unable to study the impact of lifetime alcohol exposure and intensity and type of physical activity (leisure vs. occupational). In addition, we did not have sufficient power for beverage-specific analysis due to the relatively low alcohol intake in our study population. Previous studies have shown no difference by type of alcoholic beverage (i.e. beer, wine, liquor) on breast cancer risk [19], [47], [48], but no studies to date have been large enough to study beverage-specific effects on mammographic density. We were also unable to retrieve mammograms for all eligible participants. Although women with mammograms were slightly different from those without in terms of reproductive and hormonal factors, differences were generally small and did not reflect a specific lifestyle behavior. Differential misclassification is also very unlikely given the fact that women are unaware of their breast density. Furthermore, we were able to replicate well-known associations with age, BMI and reproductive factors, which can be interpreted as an internal validation of our study.

Our findings indicate that increasing alcohol intake is associated with an increase in mammography density, whereas smoking and physical activity do not seem to influence density. The observed interaction between alcohol and HRT use suggests that HRT users may consider lowering their alcohol intake in order to reduce their mammographic density and breast cancer risk.

## References

[1] McCormack VA, dos Santos Silva I (2006) Breast density and parenchymal patterns as markers of breast cancer risk: A meta-analysis. Cancer Epidemiol Biomarkers Prev 15: 1159–1169 10.1158/1055–9965.EPI-06-0034. 16775176 10.1158/1055-9965.EPI-06-0034

[2] Cabanes A, Pastor-Barriuso R, Garcia-Lopez M, Pedraz-Pingarron C, Sanchez-Contador C, et al (2011) Alcohol, tobacco, and mammographic density: A population-based study. Breast Cancer Res Treat 129: 135–147 10.1007/s10549-011-1414-5; 10.1007/s10549-011-1414-5. 21373874 10.1007/s10549-011-1414-5

[3] Yaghjyan L, Mahoney MC, Succop P, Wones R, Buckholz J, et al (2012) Relationship between breast cancer risk factors and mammographic breast density in the fernald community cohort. Br J Cancer 106: 996–1003 10.1038/bjc.2012.1; 10.1038/bjc.2012.1. 22281662 10.1038/bjc.2012.1 PMC3305977

[4] Masala G, Ambrogetti D, Assedi M, Giorgi D, Del Turco MR, et al (2006) Dietary and lifestyle determinants of mammographic breast density. A longitudinal study in a mediterranean population. Int J Cancer 118: 1782–1789 10.1002/ijc.21558. 16231317 10.1002/ijc.21558

[5] Maskarinec G, Takata Y, Pagano I, Lurie G, Wilkens LR, et al (2006) Alcohol consumption and mammographic density in a multiethnic population. Int J Cancer 118: 2579–2583 10.1002/ijc.21705. 16380998 10.1002/ijc.21705

[6] Vachon CM, Kushi LH, Cerhan JR, Kuni CC, Sellers TA (2000) Association of diet and mammographic breast density in the minnesota breast cancer family cohort. Cancer Epidemiol Biomarkers Prev 9: 151–160. 10698475

[7] Qureshi SA, Couto E, Hofvind S, Wu AH, Ursin G (2012) Alcohol intake and mammographic density in postmenopausal norwegian women. Breast Cancer Res Treat 131: 993–1002 10.1007/s10549-011-1812-8; 10.1007/s10549-011-1812-8. 21993860 10.1007/s10549-011-1812-8

[8] Gapstur SM, Lopez P, Colangelo LA, Wolfman J, Van Horn L, et al (2003) Associations of breast cancer risk factors with breast density in hispanic women. Cancer Epidemiol Biomarkers Prev 12: 1074–1080. 14578145

[9] Sala E, Warren R, Duffy S, Welch A, Luben R, et al (2000) High risk mammographic parenchymal patterns and diet: A case-control study. Br J Cancer 83: 121–126 10.1054/bjoc.2000.1151. 10883679 10.1054/bjoc.2000.1151 PMC2374534

[10] Bremnes Y, Ursin G, Bjurstam N, Gram IT (2007) Different measures of smoking exposure and mammographic density in postmenopausal norwegian women: A cross-sectional study. Breast Cancer Res 9: R73 10.1186/bcr1782. 17963507 10.1186/bcr1782 PMC2242671

[11] Modugno F, Ngo DL, Allen GO, Kuller LH, Ness RB, et al (2006) Breast cancer risk factors and mammographic breast density in women over age 70. Breast Cancer Res Treat 97: 157–166 10.1007/s10549-005-9105-8. 16362132 10.1007/s10549-005-9105-8

[12] Vachon CM, Kuni CC, Anderson K, Anderson VE, Sellers TA (2000) Association of mammographically defined percent breast density with epidemiologic risk factors for breast cancer (united states). Cancer Causes Control 11: 653–662. 10977110 10.1023/a:1008926607428

[13] Qureshi SA, Ellingjord-Dale M, Hofvind S, Wu AH, Ursin G (2012) Physical activity and mammographic density in a cohort of postmenopausal norwegian women; a cross-sectional study. Springerplus 1: 75 10.1186/2193-1801-1-75. 23397025 10.1186/2193-1801-1-75 PMC3565086

[14] Masala G, Assedi M, Ambrogetti D, Sera F, Salvini S, et al (2009) Physical activity and mammographic breast density in a mediterranean population: The EPIC florence longitudinal study. Int J Cancer 124: 1654–1661 10.1002/ijc.24099; 10.1002/ijc.24099. 19085933 10.1002/ijc.24099

[15] Irwin ML, Aiello EJ, McTiernan A, Baumgartner RN, Baumgartner KB, et al (2006) Pre-diagnosis physical activity and mammographic density in breast cancer survivors. Breast Cancer Res Treat 95: 171–178 10.1007/s10549-005-9063-1. 16319988 10.1007/s10549-005-9063-1 PMC3034407

[16] Yaghjyan L, Colditz GA, Wolin K (2012) Physical activity and mammographic breast density: A systematic review. Breast Cancer Res Treat 135: 367–380 10.1007/s10549-012-2152-z; 10.1007/s10549-012-2152-z. 22814722 10.1007/s10549-012-2152-z PMC3641148

[17] Luo J, Horn K, Ockene JK, Simon MS, Stefanick ML, et al (2011) Interaction between smoking and obesity and the risk of developing breast cancer among postmenopausal women: The women's health initiative observational study. Am J Epidemiol 174: 919–928 10.1093/aje/kwr192; 10.1093/aje/kwr192. 21878422 10.1093/aje/kwr192 PMC3218630

[18] Slattery ML, Edwards S, Murtaugh MA, Sweeney C, Herrick J, et al (2007) Physical activity and breast cancer risk among women in the southwestern united states. Ann Epidemiol 17: 342–353 10.1016/j.annepidem.2006.10.017. 17462544 10.1016/j.annepidem.2006.10.017 PMC2925501

[19] Chen WY, Colditz GA, Rosner B, Hankinson SE, Hunter DJ, et al (2002) Use of postmenopausal hormones, alcohol, and risk for invasive breast cancer. Ann Intern Med 137: 798–804. 12435216 10.7326/0003-4819-137-10-200211190-00008

[20] Nielsen NR, Gronbaek M (2008) Interactions between intakes of alcohol and postmenopausal hormones on risk of breast cancer. Int J Cancer 122: 1109–1113 10.1002/ijc.23195. 17966122 10.1002/ijc.23195

[21] Magnusson C, Baron J, Persson I, Wolk A, Bergstrom R, et al (1998) Body size in different periods of life and breast cancer risk in post-menopausal women. Int J Cancer 76: 29–34. 9533758 10.1002/(sici)1097-0215(19980330)76:1<29::aid-ijc6>3.0.co;2-#

[22] Eriksson L, Czene K, Rosenberg L, Humphreys K, Hall P (2012) The influence of mammographic density on breast tumor characteristics. Breast Cancer Res Treat 134: 859–866 10.1007/s10549-012-2127-0; 10.1007/s10549-012-2127-0. 22710708 10.1007/s10549-012-2127-0

[23] Boyd N, Martin L, Stone J, Little L, Minkin S, et al (2002) A longitudinal study of the effects of menopause on mammographic features. Cancer Epidemiol Biomarkers Prev 11: 1048–1053. 12376506

[24] Byng JW, Boyd NF, Fishell E, Jong RA, Yaffe MJ (1994) The quantitative analysis of mammographic densities. Phys Med Biol 39: 1629–1638. 15551535 10.1088/0031-9155/39/10/008

[25] Sterne JA, White IR, Carlin JB, Spratt M, Royston P, et al (2009) Multiple imputation for missing data in epidemiological and clinical research: Potential and pitfalls. BMJ 338: b2393 10.1136/bmj.b2393. 19564179 10.1136/bmj.b2393 PMC2714692

[26] Royston P (2009) Multiple imputation of missing values: Further update of ice, with an emphasis on categorical variables. Stata Journal 9: 466–477.

[27] Rutter CM, Mandelson MT, Laya MB, Seger DJ, Taplin S (2001) Changes in breast density associated with initiation, discontinuation, and continuing use of hormone replacement therapy. JAMA 285: 171–176. 11176809 10.1001/jama.285.2.171

[28] Hamajima N, Hirose K, Tajima K, Rohan T, Calle EE, et al (2002) Alcohol, tobacco and breast cancer--collaborative reanalysis of individual data from 53 epidemiological studies, including 58,515 women with breast cancer and 95,067 women without the disease. Br J Cancer 87: 1234–1245 10.1038/sj.bjc.6600596. 12439712 10.1038/sj.bjc.6600596 PMC2562507

[29] Singletary KW, Gapstur SM (2001) Alcohol and breast cancer: Review of epidemiologic and experimental evidence and potential mechanisms. JAMA 286: 2143–2151. 11694156 10.1001/jama.286.17.2143

[30] Zhang SM, Lee IM, Manson JE, Cook NR, Willett WC, et al (2007) Alcohol consumption and breast cancer risk in the women's health study. Am J Epidemiol 165: 667–676 10.1093/aje/kwk054. 17204515 10.1093/aje/kwk054

[31] Li CI, Chlebowski RT, Freiberg M, Johnson KC, Kuller L, et al (2010) Alcohol consumption and risk of postmenopausal breast cancer by subtype: The women's health initiative observational study. J Natl Cancer Inst 102: 1422–1431 10.1093/jnci/djq316; 10.1093/jnci/djq316. 20733117 10.1093/jnci/djq316 PMC2943525

[32] Narod SA (2011) Hormone replacement therapy and the risk of breast cancer. Nat Rev Clin Oncol 8: 669–676 10.1038/nrclinonc.2011.110; 10.1038/nrclinonc.2011.110. 21808267 10.1038/nrclinonc.2011.110

[33] Onland-Moret NC, Peeters PH, van der Schouw YT, Grobbee DE, van Gils CH (2005) Alcohol and endogenous sex steroid levels in postmenopausal women: A cross-sectional study. J Clin Endocrinol Metab 90: 1414–1419 10.1210/jc.2004-0614. 15572431 10.1210/jc.2004-0614

[34] Colditz GA (1998) Relationship between estrogen levels, use of hormone replacement therapy, and breast cancer. J Natl Cancer Inst 90: 814–823. 9625169 10.1093/jnci/90.11.814

[35] Dorgan JF, Baer DJ, Albert PS, Judd JT, Brown ED, et al (2001) Serum hormones and the alcohol-breast cancer association in postmenopausal women. J Natl Cancer Inst 93: 710–715. 11333294 10.1093/jnci/93.9.710

[36] Ginsburg ES, Mello NK, Mendelson JH, Barbieri RL, Teoh SK, et al (1996) Effects of alcohol ingestion on estrogens in postmenopausal women. JAMA 276: 1747–1751. 8940324 10.1001/jama.1996.03540210055034

[37] Lands WE, Zakhari S (1991) The case of the missing calories. Am J Clin Nutr 54: 47–48. 2058586 10.1093/ajcn/54.1.47

[38] Westerterp KR (2004) Diet induced thermogenesis. Nutr Metab (Lond) 1: 5 10.1186/1743-7075-1-5. 15507147 10.1186/1743-7075-1-5 PMC524030

[39] Shepherd JA, Kerlikowske K (2012) Do fatty breasts increase or decrease breast cancer risk? Breast Cancer Res 14: 102 10.1186/bcr3081. 22277587 10.1186/bcr3081 PMC3496115

[40] Butler LM, Gold EB, Conroy SM, Crandall CJ, Greendale GA, et al (2010) Active, but not passive cigarette smoking was inversely associated with mammographic density. Cancer Causes Control 21: 301–311 10.1007/s10552-009-9462-4; 10.1007/s10552-009-9462-4. 19915951 10.1007/s10552-009-9462-4 PMC2810361

[41] Xue F, Willett WC, Rosner BA, Hankinson SE, Michels KB (2011) Cigarette smoking and the incidence of breast cancer. Arch Intern Med 171: 125–133 10.1001/archinternmed.2010.503; 10.1001/archinternmed.2010.503. 21263102 10.1001/archinternmed.2010.503 PMC3131146

[42] Wu Y, Zhang D, Kang S (2013) Physical activity and risk of breast cancer: A meta-analysis of prospective studies. Breast Cancer Res Treat 137: 869–882 10.1007/s10549-012-2396-7; 10.1007/s10549-012-2396-7. 23274845 10.1007/s10549-012-2396-7

[43] Peters TM, Ekelund U, Leitzmann M, Easton D, Warren R, et al (2008) Physical activity and mammographic breast density in the EPIC-norfolk cohort study. Am J Epidemiol 167: 579–585 10.1093/aje/kwm350. 18162477 10.1093/aje/kwm350

[44] McTiernan A, Tworoger SS, Ulrich CM, Yasui Y, Irwin ML, et al (2004) Effect of exercise on serum estrogens in postmenopausal women: A 12-month randomized clinical trial. Cancer Res 64: 2923–2928. 15087413 10.1158/0008-5472.can-03-3393

[45] Siozon CC, Ma H, Hilsen M, Bernstein L, Ursin G (2006) The association between recreational physical activity and mammographic density. Int J Cancer 119: 1695–1701 10.1002/ijc.22020. 16688715 10.1002/ijc.22020

[46] Reeves KW, Gierach GL, Modugno F (2007) Recreational physical activity and mammographic breast density characteristics. Cancer Epidemiol Biomarkers Prev 16: 934–942 10.1158/1055–9965.EPI-06-0732. 17507619 10.1158/1055-9965.EPI-06-0732

[47] Smith-Warner SA, Spiegelman D, Yaun SS, van den Brandt PA, Folsom AR, et al (1998) Alcohol and breast cancer in women: A pooled analysis of cohort studies. JAMA 279: 535–540. 9480365 10.1001/jama.279.7.535

[48] Li Y, Baer D, Friedman GD, Udaltsova N, Shim V, et al (2009) Wine, liquor, beer and risk of breast cancer in a large population. Eur J Cancer 45: 843–850 10.1016/j.ejca.2008.11.001; 10.1016/j.ejca.2008.11.001. 19095438 10.1016/j.ejca.2008.11.001

